# Mobitz I Atrioventricular Block Following Combined Opioid and Benzodiazepine Overdose: A Case Report

**DOI:** 10.7759/cureus.104528

**Published:** 2026-03-02

**Authors:** Mariana Valdez Thomas, Michelle Carrasquel, Maria del Pilar Acosta, Ynsnardy J Hurtado-Leon, Michael Mandel

**Affiliations:** 1 Internal Medicine, Montefiore Medical Center, New Rochelle Campus, New Rochelle, USA; 2 Internal Medicine, McLaren Greater Lansing, Lansing, USA

**Keywords:** atrioventricular block, benzodiazepine overdose, bradycardia, conduction disturbance, mobitz i, opioid overdose

## Abstract

Opioids and benzodiazepines are well recognized for their depressant effects on the central nervous system, most notably respiratory depression, but atrioventricular (AV) block is an uncommon manifestation.

We present the case of a 35-year-old man with polysubstance use disorder who was admitted following a combined fentanyl-benzodiazepine overdose. On admission, he was somnolent but arousable, with bradycardia and oxygen desaturation. Electrocardiogram (ECG) demonstrated Mobitz I second-degree AV block with progressive PR interval prolongation and intermittent non-conducted P waves. Laboratory evaluation revealed mild metabolic acidosis without significant electrolyte abnormalities, and toxicology was positive for fentanyl and benzodiazepines. Supportive management, including intravenous fluids, oxygen, and naloxone, led to gradual improvement. Telemetry demonstrated recovery from Mobitz I block to first-degree AV block, and ultimately restoration of normal sinus rhythm within 48 hours. Echocardiogram showed preserved left ventricular ejection fraction and no structural abnormalities.

This case highlights a rare presentation of reversible Mobitz I AV block following mixed opioid-benzodiazepine intoxication. Recognition of Wenckebach as a transient conduction disturbance, consistent with guideline-directed management, prevented unnecessary pacemaker implantation. Careful monitoring is essential to detect dynamic conduction changes in drug-induced cardiac toxicity.

## Introduction

Opioid misuse represents a major global public health crisis, with synthetic opioids such as fentanyl accounting for a substantial proportion of overdose-related morbidity and mortality worldwide [1,2]. In the United States, fentanyl has emerged as a leading contributor to overdose-related deaths, responsible for more than 70,000 fatalities annually [2]. While respiratory depression remains the most recognized manifestation of opioid toxicity, cardiovascular complications, including bradycardia, hypotension, and cardiac conduction abnormalities, are increasingly recognized [3-5].

Opioids exert electrophysiologic effects through autonomic modulation and direct ion channel interactions, resulting in increased vagal tone and suppression of sinoatrial and atrioventricular (AV) nodal conduction [3-5]. Benzodiazepines may further potentiate parasympathetic tone and reduce sympathetic drive, increasing susceptibility to bradyarrhythmias when used concurrently [6]. Although high-grade AV block has been described in opioid toxicity, transient Mobitz I AV block remains less well characterized in this setting [7,8].

Recognition of reversible conduction abnormalities is critical because guideline-directed management recommends conservative therapy when drug toxicity is suspected [9]. We present a case of reversible Mobitz I AV block following fentanyl-benzodiazepine intoxication, highlighting the importance of systematic diagnostic evaluation, continuous cardiac monitoring, and avoidance of unnecessary pacing interventions.

## Case presentation

A 35-year-old Hispanic man with a history of polysubstance use disorder and depression was found unresponsive at home by family members and transported to the Emergency Department. He was unemployed and lived with relatives who provided intermittent supervision and social support. His past medical history was notable only for depression treated with sertraline 50 mg daily. He had no known structural heart disease, prior arrhythmias, syncope, thyroid disorders, obstructive sleep apnea, or cardiovascular conditions. He was not taking any AV nodal blocking agents, including beta-blockers, calcium channel blockers, digoxin, or antiarrhythmic medications. He denied use of over-the-counter supplements or herbal medications. He had no known drug allergies.

His substance use history included chronic recreational use of fentanyl and benzodiazepines, primarily via intranasal and intravenous routes. According to family members, he had used fentanyl and alprazolam approximately two to four hours prior to being found unresponsive. The exact dose and route of ingestion could not be confirmed.

On arrival, the patient was somnolent but arousable to verbal stimuli. Vital signs included blood pressure 107/83 mmHg, heart rate 48 beats per minute, respiratory rate 14 breaths per minute, oxygen saturation 91% on room air, and temperature 36.5°C. Physical examination revealed miotic pupils and sinus bradycardia without murmurs, rubs, or gallops.

**Figure 1:**
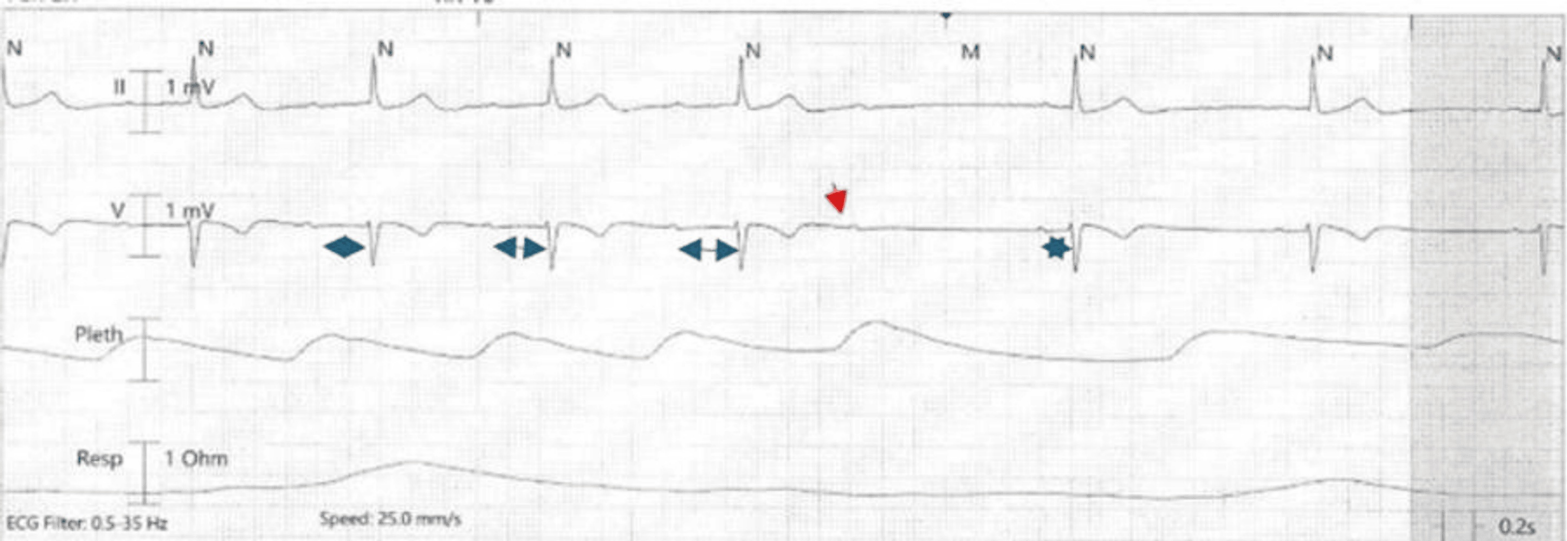
Telemetry tracing demonstrating Mobitz type I (Wenckebach) atrioventricular block, with progressive PR interval prolongation (blue double arrows) culminating in a non-conducted P wave (red arrow), followed by a junctional escape beat (blue star).

Initial 12-lead electrocardiogram (ECG) demonstrated Mobitz I second-degree AV block, characterized by progressive PR interval prolongation and intermittent non-conducted P waves (Figure 1). Continuous telemetry monitoring confirmed Mobitz I AV block.

Laboratory evaluation demonstrated mild metabolic acidosis, with normal serum potassium, magnesium, calcium, and thyroid-stimulating hormone levels. Serial cardiac troponin levels were negative, effectively excluding myocardial ischemia. There were no clinical features suggestive of myocarditis (Table 1).

**Table 1:** Laboratory values.

Test	Patient value	Reference range
pH (arterial blood gas)	7.31	7.35-7.45
HCO₃⁻ (mmol/L)	20	22-28
Sodium (mmol/L)	138	135-145
Potassium (mmol/L)	4.1	3.5-5.0
Chloride (mmol/L)	101	98-107
Creatinine (mg/dL)	0.9	0.6-1.3
Glucose (mg/dL)	106	70-110

Urine toxicology screening, using qualitative immunoassay, was positive for fentanyl and benzodiazepines and negative for cocaine, amphetamines, methadone, cannabinoids, and other cardiotoxic substances. Serum ethanol level was undetectable.

**Figure 2:**
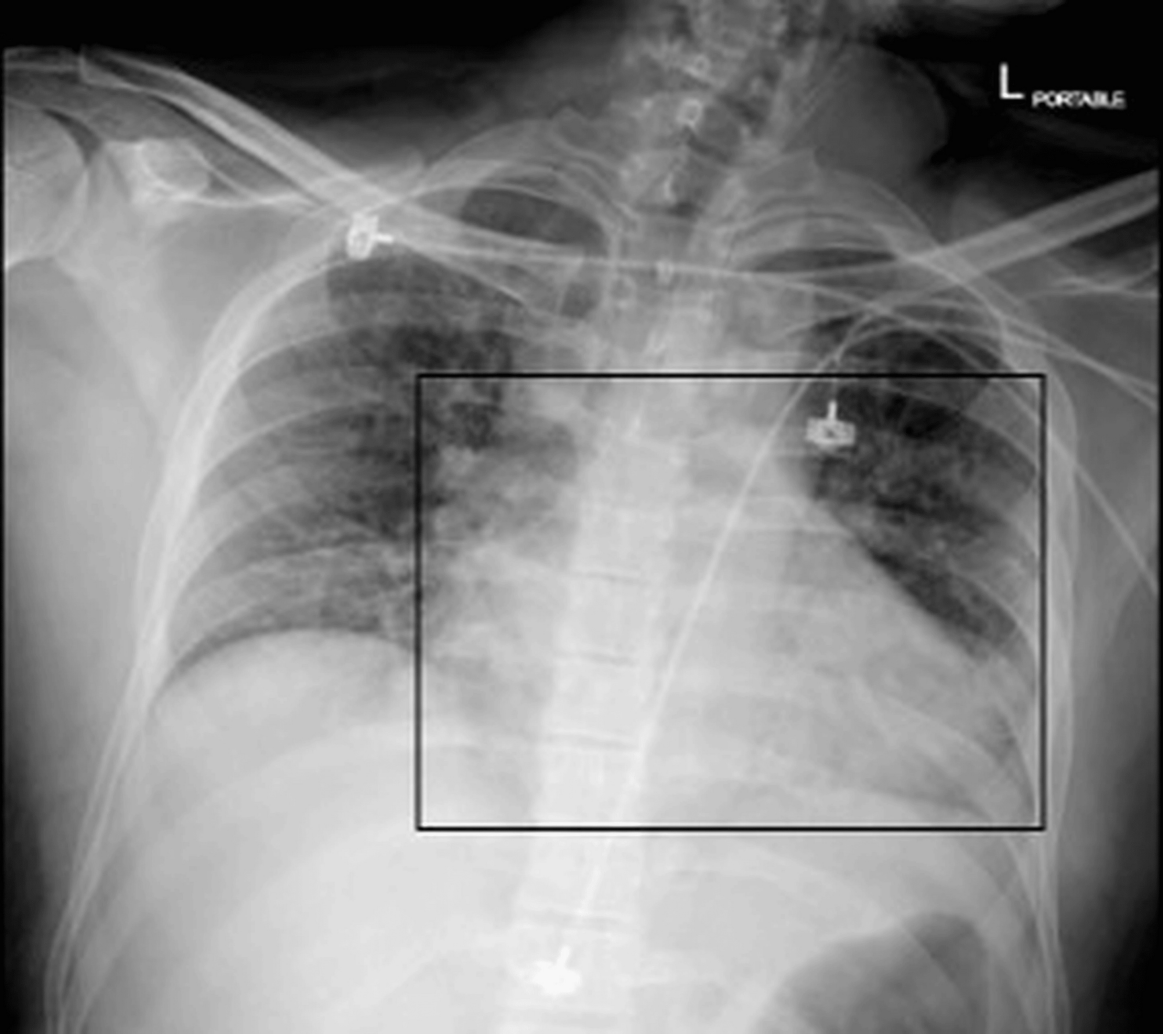
Chest radiograph (anteroposterior view) showing a moderately enlarged cardiac silhouette, without pulmonary infiltrates (rectangle highlights cardiomegaly).

**Figure 3:**
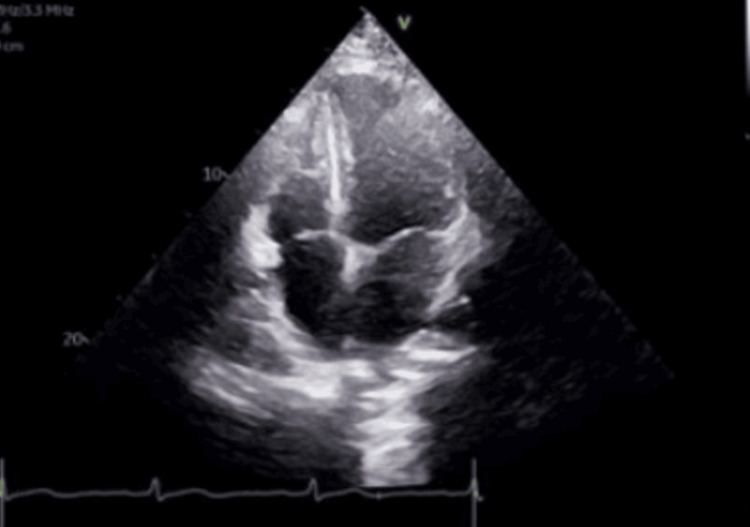
Transthoracic echocardiogram (apical four‑chamber view) demonstrating preserved biventricular size and function, with a left ventricular ejection fraction of 55%-60%.

Chest radiography was obtained as a portable anteroposterior projection and demonstrated a mildly enlarged cardiac silhouette (Figure 2). However, transthoracic echocardiography demonstrated normal cardiac chamber size, preserved left ventricular systolic function with an ejection fraction of 55%-60%, normal right ventricular size and function, no regional wall motion abnormalities, and trivial mitral regurgitation without structural abnormalities (Figure 3).

Supportive management included supplemental oxygen via nasal cannula and intravenous normal saline. Naloxone 0.4 mg was administered intravenously, resulting in improvement in mental status and respiratory function. Continuous telemetry monitoring was maintained. Cardiology consultation recommended conservative management, consistent with guideline-directed therapy for reversible AV block.

Telemetry demonstrated Mobitz I AV block during the first 12 hours, improvement to first-degree AV block by 24 hours, and complete resolution, with restoration of normal sinus rhythm by 48 hours.

**Figure 4:**
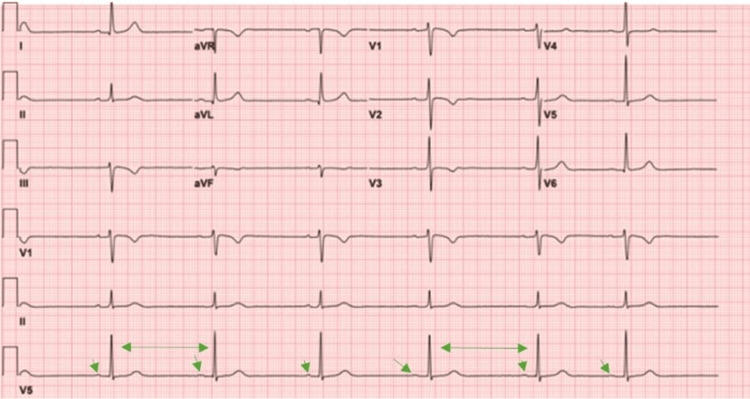
Electrocardiogram (ECG) after 48 hours of observation, demonstrating restoration of normal sinus rhythm with 1:1 atrioventricular (AV) conduction (green arrow).

The patient was discharged on hospital day 3. At two-week follow-up, he remained asymptomatic, with normal ECG findings (Figure 4).

## Discussion

Opioids such as fentanyl exert electrophysiologic effects through increased vagal tone and direct ion channel modulation, resulting in suppression of AV nodal conduction [3-5]. Benzodiazepines may further augment parasympathetic tone and reduce sympathetic drive, contributing to bradyarrhythmias [6]. The causal relationship in this case is supported by temporal association, systematic exclusion of alternative etiologies, and consistency with existing literature. Drug-induced AV block frequently resolves following removal of the offending agent [7,8]. Guidelines from the American College of Cardiology, American Heart Association, and Heart Rhythm Society recommend against permanent pacing in AV block caused by reversible etiologies, such as drug toxicity [9]. Naloxone reverses opioid-mediated autonomic suppression and improves conduction abnormalities by restoring sympathetic tone [10]. Limitations include a lack of quantitative toxicology and the inability to definitively exclude all co-ingestants.

## Conclusions

Mobitz I AV block can occur in the setting of opioid and benzodiazepine intoxication. Alternative causes, including ischemia, structural heart disease, electrolyte abnormalities, and thyroid dysfunction, were excluded. Echocardiography confirmed structurally normal cardiac anatomy. Recognition of reversible causes is essential to avoid unnecessary pacing interventions and ensure appropriate guideline-directed management.
